# Analysis of the use of central venous catheters, mortality and catheter-related bloodstream infections in patients admitted to the ICU and after discharge to ward

**DOI:** 10.1186/2197-425X-3-S1-A891

**Published:** 2015-10-01

**Authors:** C Martín, JA Silva, JE Romo, LA Barcena, S Saboya, C Marian

**Affiliations:** Hospital General Universitario de Guadalajara, Guadalajara, Spain; Hospital Universitario Puerta de Hierro, Majadahonda, Spain

## Objectives

Analyze the number and indication for insertion of central venous catheters (CVCs), the incidence of catheter-related bloodstream infection (CRBSI) and the mortality in the intensive care unit (ICU), and after discharge to ward, in patients requiring CVCs in the ICU.

## Methods

Retrospective, descriptive analysis, from October 2013 to December 2014, of all patients admitted to the ICU. We considered: age, gender, Apache II, indication for canalization of CVC, number of CVCs, days in ICU with CVC, CRBSI and mortality in ICU and after discharge to ward, if at the time of discharge patients were carrying CVC.

## Results

590 admitted patients: age 64.4 ± 15.4 years, 65% males, Apache II14.6 ± 9.2. 54.4% (321/590) required CVC. Apache II - with CVC 19.2 ± 9.4 vs 9.1 ± 5 without CVC (p < 0.001). One CVC was inserted in 231 (72%), 2 in 72 (22.4%), 3 in 14 (4.4%), 4 in 2 (0.6%) and 5 in another 2 (0.6%). A total of 435 CVCs were inserted: 40% subclavian, 31.7% jugular, 26.9% femoral and 1.4% peripherally inserted central catheters.The main reason for CVCs was: sedation 68.8% (221), catecholamines 65.1% (209), either 83% (266) or both 51.1% (164); other causes: insulin infusion 15.9% (51), parenteral feeding 12.8% (41), continuous renal replacement therapy 7.5% (24), other 6.5% (21).The total CVC days in the ICU was 2545; 1 CRBSI episode was diagnosed (CRBSI incidence density 0.4 episodes/1000 catheter days). The total number of days of stay in the ICU was 2805.95 days (incidence density 0.36 episodes/1000 income days). CVC utilization ratio: 0.91.For patients requiring CVC, 43% (116/270) were discharged to ward with CVC. In ward, CRBSI incidence density was 0.29 episodes/1000 catheter days in hospital after ICU (one patient in 3476 days). We could not specify the number of days with CVC after discharge from ICU.

Mortality analysis showed:

1) In ICU: patients with CVC 15.9% (51/321) vs without CVC 1.5% (4/269), p < 0.001; OR 12.5 (95% CI 4.5-35.1); (Apache II 28.3 ± 9.5 vs 20.5 ± 18.4, p = 0.148 NS).

2) After discharge from ICU: patients with CVC 8.6% (10/116) vs without CVC 2.6% (11/419); p = 0.003 (OR 3.5, 95% CI 1.4-8.5); Apache II 16.87 ± 84 vs 12.23 ± 7.5 (p < 0.001).

## Conclusions

In our study, more than half of the patients admitted to the ICU pointed CVCs.

72% needed only one CVC, subclavian or jugular (71.7%), mainly for using catecholamines or sedatives. In the ICU, CRBSI incidence density was 0.4/1000 catheter days. This rate could not be clarified after discharge to ward. The mortality in ICU and after discharge to ward was higher in more seriously ill patients and those with CVC, although none died of CRBSI.

